# Hard Carbons as Anodes in Sodium-Ion Batteries: Sodium Storage Mechanism and Optimization Strategies

**DOI:** 10.3390/molecules27196516

**Published:** 2022-10-02

**Authors:** Liyang Liu, Ye Tian, Abubakar Abdussalam, Muhammad Rehan Hasan Shah Gilani, Wei Zhang, Guobao Xu

**Affiliations:** 1 State Key Laboratory of Electroanalytical Chemistry, Changchun Institute of Applied Chemistry, Chinese Academy of Sciences, Changchun, Jilin 130022, China; 2 School of Applied Chemistry and Engineering, University of Science and Technology of China, Hefei 230026, China; 3 The College of Civil Engineering, Shenyang Urban Construction University, Shenyang, Liaoning 110167, China; 4 College of Natural and Pharmaceutical Sciences, Department of Chemistry, Bayero University, P.M.B 3011, Kano 700006, Nigeria; 5 Institute of Chemical Sciences, Bahauddin Zakariya University, Multan 60800, Pakistan

**Keywords:** hard carbon, Sodium-ion batteries, electrolyte, anode, carbon materials, modification, electrochemical performance, capacitive properties

## Abstract

Sodium-ion batteries (SIBs) are regarded as promising alternatives to lithium-ion batteries (LIBs) in the field of energy, especially in large-scale energy storage systems. Tremendous effort has been put into the electrode research of SIBs, and hard carbon (HC) stands out among the anode materials due to its advantages in cost, resource, industrial processes, and safety. However, different from the application of graphite in LIBs, HC, as a disordered carbon material, leaves more to be completely comprehended about its sodium storage mechanism, and there is still plenty of room for improvement in its capacity, rate performance and cycling performance. This paper reviews the research reports on HC materials in recent years, especially the research process of the sodium storage mechanism and the modification and optimization of HC materials. Finally, the review summarizes the sterling achievements and the challenges on the basis of recent progress, as well as the prospects on the development of HC anode materials in SIBs.

## 1. Introduction

There is accumulating evidence of over two centuries of fossil fuel burning. Politicians and business leaders around the world have continued to reiterate their concerns over the excessive carbon dioxide emissions that are considered a serious problem. The world is urgently facing an adjustment of the energy framework without delay; it is therefore preferable to switch to electrification. The research and development of rechargeable batteries has begun to play more and more of an essential role in order to achieve a sustainable energy society. From the electronic vehicle to large-scale energy storage, the rechargeable battery field is of great importance. Since lithium-ion batteries (LIBs) were approved for portable device applications and achieved basic commercialization from the 1990s [1], LIBs have been considered the energy technology of choice, amenable to continuous improvement and advances, and paving ways for emerging applications [2,3].

While tremendous efforts are devoted to developing higher power and energy performance in order to build a grid-scale energy storage system (ESS) incorporated with advanced clean energy, such as wind and solar energy [4], LIBs have become promising candidates for this great blueprint. However, there is a lot to consider when it comes to large-scale industrialization in view of the abnormally high price of lithium, the increasing cost of other LIB materials, and the rarity of lithium resources [5,6]. Hence, the establishment of simple, innovative, economically feasible new battery materials for large-scale energy storage is in great demand. For large power systems, the focus shifts to production cost while maintaining performance benefits. It is becoming more popular to obtain excellent low-cost battery materials from abundant resources. In the context above, sodium, as the second lightest alkali metal next to lithium, is abundant in Earth’s crust and sea. Due to its properties being close to lithium as an electrochemical battery material, and having similar industrial processes, sodium has recently received considerable attention as an alternative material from which to develop batteries [7,8].

Sodium-ion batteries (SIBs) are considered “beyond Li-ion” batteries and have gained substantial progress since the 2010s. Together with K-ion batteries, a review [9] compared SIBs with LIBs, all-solid-state batteries and multivalent batteries, with a section focusing on cost and performance. A breakthrough has been made in the research of cathode materials for SIBs, which showed an average operating voltage of 3.95 V and a capacity of 128 mAh g^−1^, delivering a power density of about 507 Ah kg^−1^ [10]. This kind of Na_3_V_2_(PO_4_)_2_F_3_(NVPF) as an SIB cathode material is comparable to LIB electrodes, such as LiMn_2_O_4_ [11].

**Figure 1:**
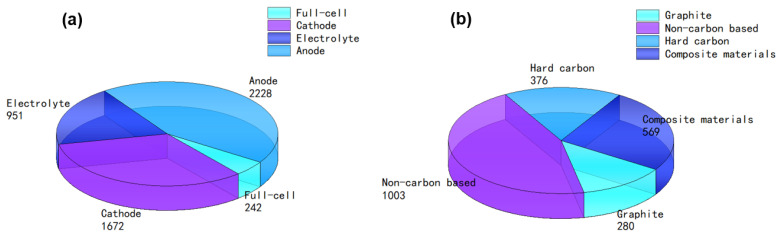
Rough count number of studies published in the last five years (obtained from Web of Science core-collection database, from 2018 and data up to August in 2022). (**a**) The direction of studies published in the SIB field (left). (**b**) Various materials studies published in the anodic direction (right).

The research and development of SIBs mainly include cathode, anode, electrolyte, and other parts, which involve a diaphragm, binder, and additives. Among them, cathode material is the most studied, and anode material is the second. Cathode materials include various kinds of layered oxides [12], polyanionic cathodes [13] and Prussian blue cathodes [14], which account for about 40% of the total cost of batteries. From the full-cell point of view, the development of anode materials is also crucial, depending on the kinds of materials used. For example, titanium-based materials [15,16], metal oxides [17,18], metallic composite materials [19,20,21], organic materials [22,23], and carbon-based materials [24,25,26,27] have all been used as anodes. The electrochemical properties of different materials are illustrated in Table 1 below (a rough number of studies published in the last five years as shown in Figure 1a). Among all of these materials, considering cost and resource factors, carbon-based material is undoubtedly a promising choice. A pioneering breakthrough was achieved, leading to increasing electrochemical performance with the sodium storage capacity at 300.6 mAh g^−1^ and the initial Coulombic efficiency (ICE) at 88.6% [26]. Herein, we summarize the different carbon-based anode materials used in SIBs, such as graphite, soft carbon, and hard carbon (HC), which is also called non-graphitization carbon. Moreover, this review summarizes the work and progress of predecessors, the sodium storage mechanism, and the modification and advantages of HC.

## 2. Carbon Based Anode Materials

### 2.1. Graphite

Graphite is the most widely applied material in LIBs and serves as the host structure of the anode delivering a high capacity of 372 mAh g^−1^ [29]. However, it is severely limited when used in SIBs containing carbonate electrolyte, as the capacity drops to less than 50 mAh g^−1^. Nevertheless, graphite has high stability and is fully commercialized, it is dedicated to be applied in SIBs as an anode by overcoming the inability of the Sodium-ion to form graphite intercalation compounds (Na-GICs) and restricts the insertion of Na due to thermodynamic instability [30]. The main effective solutions include: (i) introducing oxygen-containing functional groups through redox reaction [31] or morphology design [32] to increase the layer spacing for stable Na-GICs formation; and (ii) using a Sodium-ion solvent co-intercalation storage mechanism combined with an ether-based electrolyte [33,34]. 

The above two schemes have contributed to the mechanism of Sodium-ion storage between graphite layers, which have also assisted when investigating and comprehending sodium storage behavior in HC materials [35,36]. However, the solvent requirement is relatively high and the reversible specific capacity is far less than that of HC, which makes the large-scale industrialization of graphite anodes difficult. For example, Wang’s group [32] synthesized an expanded graphite as a superior anode, which showed a high reversible capacity of 284 mAh g^−1^ at a current density of 20 mA g^−1^ and retained 73.92% of its capacity after 2000 cycles. 

### 2.2. Soft Carbon

With respect to amorphous carbon, including HC and soft carbon, the main difference lies in the graphitization or non-graphitization under high pyrolytic temperature. The degree of graphitization increases with a rise of pyrolytic temperature, forming a turbostratic disordered structure. Compared with HC, soft carbon tends to form large, stacked graphene layers in the stacking direction and base plane. HC, however, is characterized by a small area of graphite microcrystalline and a larger area of randomly oriented nanoscale pores even at elevated temperatures above 2800 °C.

Owing to the advantages of low-cost, adjustable layer spacing and its considerable electronic conductivity, soft carbon also shows good performance as an anode material for SIBs. Defect-rich soft carbon porous nanosheets [28], when used as an anode material, can reach a capacity of 232 mAh g^−1^ and a superior rate capability of 103 mAh g^−1^ at 1000 mA g^−1^. Li and co-workers [37] prepared soft carbon with pyrolytic anthracite as a precursor and achieved a good rate and cycling performance with a reversible capacity of 222 mAh g^−1^. Practically, soft carbon suffers from low energy density in a full battery assembling since high operating voltage can reduce the working potential. 

### 2.3. Hard Carbon

HC, also called non-graphitizing carbon, is mainly composed of short-range ordered regions of curved graphene sheets in small areas and a wealth of nanopores formed from the microcrystalline areas. The precise structure, size of the graphite microcrystalline zone, number of carbon layers, and the nanopores, are all affected by the carbonization temperature. As early as 2000, Dahn [38] first reported on the application of HC as an anode, using glucose as a carbon source to prepare pyrolytic HC, which provided a high reversible capacity of over 300 mAh g^−1^. Since then, HC as an anode has attracted tremendous attention, and different HC materials prepared by various kinds of precursors have been reported successively.

The properties of HC anodes have witnessed rapid improvements in recent years. For instance, Yang [39] prepared an N-doped HC with a soybean residue and assembled it with a Na_3_V_2_(PO_4_)_3_ cathode to show a high full-cell energy density of 146.1 Wh kg^−1^. Guo’s group [40] utilized chitin derived N-doped amorphous carbon nanofibers as an anode coordinating a Prussian blue cathode to deliver a high reversible capacity of 120 mAh g^−1^ and over 90% retention rate after 200 cycles. Although HC materials originate from low-cost and abundant resources and show excellent capacity property, HC suffers from relatively poor cycling stability and rate capability; and plenty of strategies are dedicated to reducing these disadvantages, which will be described later in the present contribution. 

## 3. Hard Carbon Materials

### 3.1. Synthetic Raw Material

It is considered that thermosetting raw materials with a strong crosslinking structure are promising precursors to generate HC materials via pyrolysis. The following materials have been used for the synthesis of HC (their specific electrochemical properties are illustrated in Table 2 below).

1. Carbohydrate or other organic polymers; for example, sucrose [41,42], cellulose [43,44], and lignin [45] have been used as precursors for the fabrication of HC anodes. In particular, Hu [44] prepared nanofibers with a short-range ordered graphite lattice and porous structures using cellulose as a raw material at the low pyrolysis temperature of 1000 °C. It exhibited a high reversible capacity of 340 mAh g^−1^.
2. Biomass source; such as peel [46,47,48], chitin [40], cotton [49], algae [50] and many others have also been used in the mass production of HC. The selection of a biomass source precursor plays an important part in the preparation of electrode materials with desired electrochemical properties [51]. According to a study reported by Xu and coauthors, an HC with a large layer spacing was fabricated based on a one-step pyrolysis of grapefruit peel in an inert atmosphere. The as-prepared HC anode has a reversible capacity of 430.5 mAh g^−1^ at a current density of 30 mA g^−1^ and remarkable cycling stability [47]. The produced HC showed a honeycomb-like structure and expanded cavities, which facilitated the diffusion of the electrolyte into the bulk of the material and shortened the distance of Sodium-ion insertion. These properties are beneficial to improve the rate capability and reversible capacity.
3. Resin carbon; for example, phenolic resin [52], and polyacrylonitrile [53] are another group of HC precursors. New progress in the research of Zhong’s group has revealed that HC can originate from a polyacrylonitrile doped polar molecule (Melamine). After spinning, they carbonized it to form HC nanofibers, which show a high gravimetric capacity, high-power capability, and long-term cycling stability of 200 mAh g^−1^ at 1 A g^−1^ current density after 1200 cycles [54].

**Table 2:** Electrochemical performance of anodes synthesized from different raw materials.

	Raw Material	Electrochemical Performance * (Capacity Performance and Cycling Stability)	Ref.
Organic polymers	Sugarcane bagasse	~290 mAh g^−1^ at 0.03 A g^−1^, 94% of capacity retention after 300 cycles.	[41]
	Sucrose	361 mAh g^−1^ at 0.02 A g^−1^, 93.4% of capacity retention after 100 cycles.	[42]
	Cellulose	~300 mAh g^−1^ at 0.1 C, an average capacity loss of 0.047%/cycle.	[43]
	Ordered cellulose nanocrystals	340 mAh g^−1^ at 0.1 A g^−1^, 88.5% of capacity retention after 400 cycles.	[44]
	Lignocellulose (peanut shell)	348 mAh g^−1^ at 0.1 C, 81.3% of capacity retention after 120 cycles.	[45]
	Mango powder	~520 mAh g^−1^ at 0.02 A g^−1^, ~204 mAh g^−1^ retained after 1000 cycles at 1 A g^−1^.	[46]
	Shaddock peel	430.5 mAh g^−1^ at 0.03 A g^−1^, 97.5% of capacity retention after 200 cycles.	[47]
Biomass source	Apricot shell	~400 mAh g^−1^ at 0.1 C, 91.9% of capacity retention after 300 cycles.	[48]
	Natural cotton	315 mAh g^−1^ at 0.1 C, 96.8% of capacity retention after 100 cycles.	[49]
	Algae	340 mAh g^−1^ at 0.025 A g^−1^, 160~170 mAh g^−1^ retained after 50 cycles.	[50]
Resin carbon	Phenolic resin	311 mAh g^−1^ at 0.02 A g^−1^, more than 80% of capacity retention after 100 cycles.	[52]
	3-aminophenol /formaldehyde resins	360 mAh g^−1^ at 0.03 A g^−1^, 86.1% of capacity retention after 100 cycles.	[53]

* Half-cell electrochemical measurements vs. Na/Na+.

### 3.2. Structure of HC

**Figure 2:**
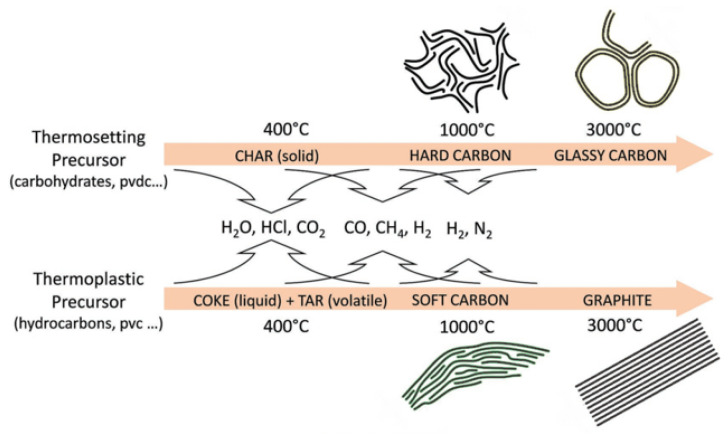
Schematic representation of the carbonization process during the pyrolysis of thermosetting and thermoplastic organic precursors [55].

Thermosetting precursors can undergo solid phase carbonization during pyrolysis. Other elements in the precursor are separated from the structure in the form of gases, and the organic molecules in the interior are fully crosslinked and hard to rearrange, making it difficult to achieve graphitization. However, the carbonaceous materials generated by thermoplastic precursors during carbonization will be affected by the residuals, such as hydrogen and oxygen, and the basic structural units of the thermoplastic precursors will rearrange to form parallel structures that are amenable to graphitization [55], as shown in Figure 2. In the case of carbohydrates, all the H and O evaporate as water, leaving a highly crosslinked network of carbons to form HC. On the contrary, in the pyrolysis process of polyvinyl chloride (PVC), H and Cl will be released in the form of hydrochloric acid gas, and the remaining hydrogen will lead to the formation of hydrocarbons, which remain at a relatively high density to benefit the graphitization and eventually form soft carbon.

**Figure 3:**
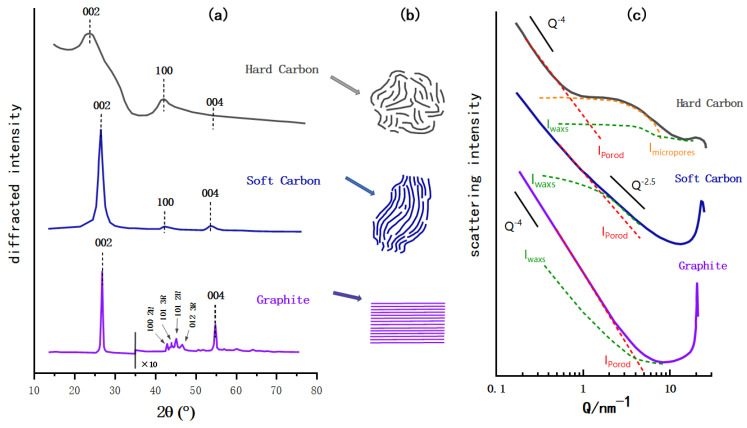
Schematic diagram of HC, soft carbon, and graphite. (a) XRD contrast diagram (modified according to the reference [56], the sharpness of the image after graphite 2θ = 35° as amplified ten times). (**b**) Schematic diagram of the microstructure of three carbon materials. (**c**) SAXs contrast diagram (modified according to the reference [57]; the I_waxs_, I_porod_ and I_micropores_ are the parameters of the structure and model established in reference).

X-ray diffraction (XRD) is utilized to investigate the crystal structures of carbon materials as shown in Figure 3a. All three carbon materials caused a distinct current signal at about 2θ = 25°, and the main XRD peak of graphite is 26.5°, which corresponds to the characteristic peak of the graphite (002) crystal face. The (002) peak of soft carbon deviates to a lower angle and is wider than that of graphite, indicating that the soft carbon forms a crystal area. Its crystallinity is not as good as graphite and the layer spacing is relatively large. Similarly, the (002) peak of HC is obviously lower in angle and larger in width, because the strong crosslinking interaction prevents the carbon layer from slipping during the pyrolysis process and generates graphite sheets with a higher degree of crystallinity. By comparing the diffraction peak (004) of the three materials, the weakening of peak intensity means a decrease of the crystallization degree. From the observation of the in-plane diffraction peak, such as the (100) peak, the angle and width of the (100) peak of soft carbon and HC are similar, which proves the similar internal turbostratic disordered structure, and the main difference between the two is the stacking degree in the c-axis direction. Compared with the (100) peak of graphite, the in-plane diffraction of amorphous carbon is weaker, indicating that more defects and bending structures may be generated in the amorphous carbon. Besides, HC and soft carbon obviously lose the peaks of (101) and (012) of in-plane diffraction, which further confirms the reduction in crystal size and disorder of the internal structure [56].

In Figure 3c, the HC was prepared at 1050 °C by the argon pyrolysis method with dehydrated sucrose as the carbon source, the soft carbon was calcined with a petroleum coke based carbon source, and the graphite was a typical synthetic graphite [57]. The small angle X-ray scattering (SAXs) patterns of the three are compared in the same schematic diagram. Under the low scattering vector, the graphite pattern is an inclined straight line, indicating that it basically has no microporous structure, and the straight line of soft carbon is slightly curved, indicating the roughness of its surface that may have a disordered structure and some micropores. In contrast, HC exhibits its characteristic pattern of a plateau in the range Q = 1~10 nm^−1^ behind the slanted line of the low scattering vector, which indicates the presence of a microporous structure [58]. 

**Figure 4:**
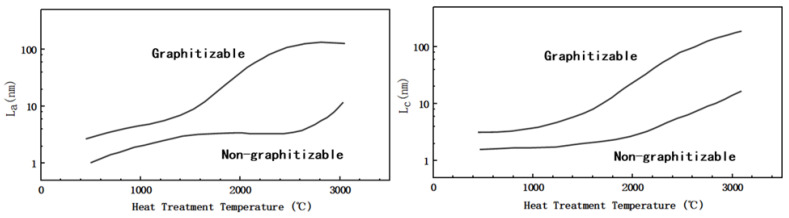
Schematic diagram of L_a_ and L_c_ of soft and HC changing with treatment temperature (modified by reference [59]).

Figure 3b shows a simple schematic diagram of the microstructure of HC, soft carbon, and graphite. As noted in the preceded sections, the structure of the amorphous carbon is quite different from that of graphite; the crystallinity, surface defects, number of micropores and graphite layer spacing vary to some extent. In addition to having the same turbostratic disordered structure as soft carbon, HC has a certain degree of crystallization, a greater degree of internal disorder, and more microporous structures. As shown in Figure 4, in terms of crystal parameters, L_a_ and L_c_ (the average width and thickness of crystals stacked along axis-a and axis-c) of HC and soft carbon are relatively close. The rise of pyrolysis temperature increased the L_a_ and L_c_ of soft carbon obviously; therefore, increasing its crystallinity and gradually turning it susceptible to graphitization. However, the change of L_a_ and L_c_ in HC is relatively gentle [59], proving that it is more difficult to undergo graphitization.

### 3.3. Sodium Storage Mechanism of HC

The research on the sodium storage mechanism of HC materials provides SIBs with several advantages in the development of a large-scale energy storage system with long-standing stability. According to previous reports, the way Sodium-ions are stored in HC materials can be through adsorption at defect-sites, edges, and heteroatoms, reversible insertion and extraction between carbon layers, and filling or adsorption in microporous regions. So far, there are several assumptions on the mechanism as follows.

#### 3.3.1. Intercalation-Adsorption

**Figure 5:**
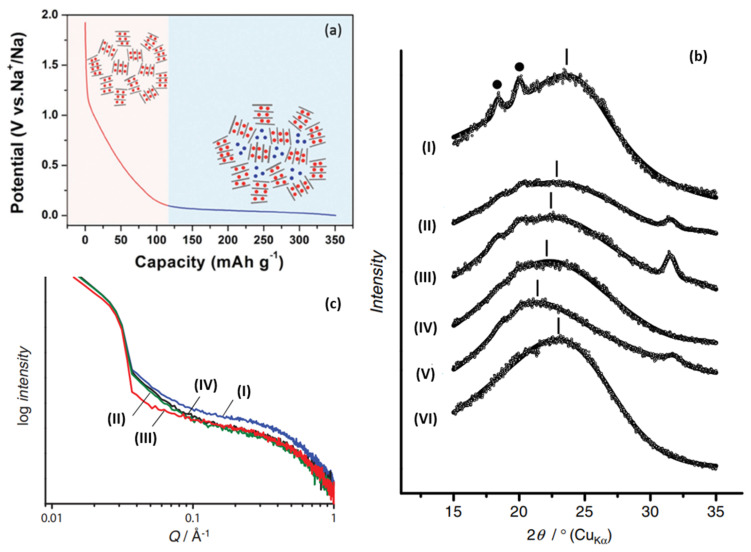
(**a**) Schematic illustration of the “intercalation-adsorption” mechanism for Sodium-ion storage in HC [63]. (**b**) Ex situ XRD patterns for HC electrodes: (I) pristine electrode, reduced in galvanostatic method to (II) 0.40 V, (III) 0.20 V, (IV) 0.10 V, (V) 0.00 V, and (VI) oxidized to 2.00 V after reduction to 0.00 V in 1 mol dm^−1^ NaClO_4_ PC. (• PVDF binder) [61]. (**c**) Ex situ SAXs patterns for HC electrodes: (I) pristine, reduced in galvanostatic method to (II) 0.20 V, (III) 0.00 V, (IV) reoxidized to 2.00 V in 1 mol dm^−1^ NaClO_4_ PC [61].

Since Dahn proposed the “House of Card” model and used it to explain the “intercalation-adsorption” mechanism, a large number of reports began to hypothesize and prove the principle of sodium storage. As shown in Figure 5a, Dahn [38] indicated that the inclined potential curve was attributed to the intercalation of sodium between parallel graphite sheets, and the insertion potential changed with the increase in Sodium-ion content, which restricted the further insertion of Sodium-ions. Moreover, the reason for the plateau area of the potential curve is the filling behavior of Sodium-ions in the microporous region, which was formed by the stacking of disorderly layers of nanocrystalline graphite (the original quote is “through a process analogous to adsorption”). In the following year, Dahn confirmed, via wide-angle in situ X-ray diffraction, that Sodium-ions can be inserted between the graphite layers of amorphous carbon [60], which led to an increase in the spacing between the graphite layers, and thus a low angle shift of the (002) peak and a decrease in its intensity. It was also proposed for the first time that the position of turbostratic stacking is distributed in a chemical environment, causing the potential curve to be inclined during the charge and discharge.

Furthermore, Komaba [61] confirmed the correlation between a graphite layer spacing and Sodium-ion intercalation (change of 002 peak in width and angle), as shown in Figure 5b. Raman spectroscopy was also used to investigate the mechanism; the electron orbital of π-bonding was affected when an Sodium-ion was intercalated into the graphite layer, which will lead to the change of C–C bond strength. Therefore, the red shift of G peak at the sloping potential region proved the intercalation of Sodium-ion into the graphite layer. In addition, SAXs were also applied, and the sodium storage behavior at the low-potential platform was accompanied by a decrease in the electron density of the micropores, which was attributed to the filling of Sodium-ions in the microporous region, as shown in Figure 5c. From the various characterization results, larger layer spacing and lower graphitization increase sodium intercalation (at an inclined potential curve), while porosity greatly affects the adsorption capacity (at a low potential plateau) [62].

#### 3.3.2. Adsorption-Intercalation

**Figure 6:**
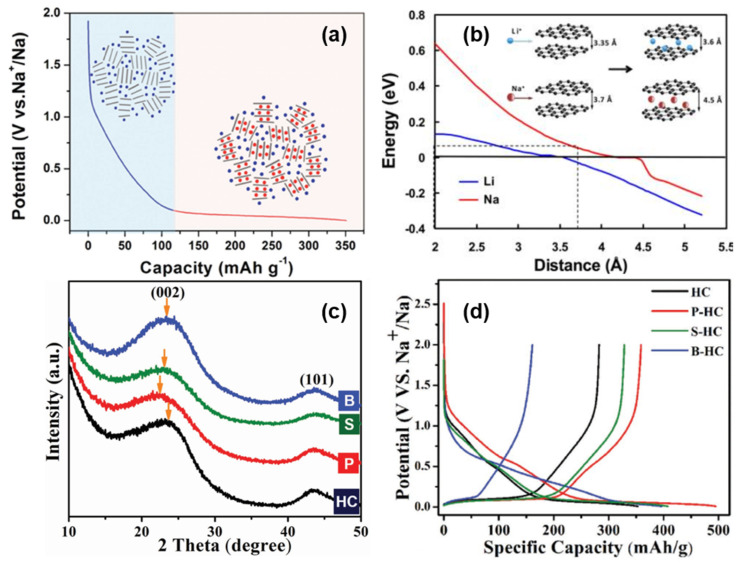
(**a**) Schematic illustration of the “adsorption-intercalation “ mechanism for Sodium-ion storage in HC [63]. (**b**) Theoretical energy cost for Na (red curve) and Li (blue curve) ion insertion into carbon as a function of the carbon interlayer distance. The inset illustrates the mechanism of Na and Li-ion insertion into carbon [64]. (**c**) XRD patterns of doped and original HCs. After P-doping and S-doping, the (002) peak shifts to a lower angle, which indicates a larger d-spacing. However, B-doping barely shifts the peak. (**d**) The galvanostatic sodiation and desodiation potential profiles of the original HC and doped HCs at a current rate of 20 mA g^−1^ [65].

In 2012, Cao’s group [36] proposed a sodium storage mechanism that is completely opposite to the “intercalation-adsorption”, as shown in Figure 6a. In order to elucidate the mechanism of Sodium-ion intercalation in HC electrodes, the energy change of Sodium-ion intercalation into HC, with the change of carbon layer spacing, was simulated theoretically. It was found that the energy variation with the change of a carbon layer spacing was relatively smooth at 0.37 nm, and the intercalation of Sodium-ion at the low potential platform of 0~0.1 V was very similar to that of graphite lithium storage. Therefore, it is inferred that the low potential platform corresponds to the reversible insertion and extraction in NaC_x_ formation (shown in Figure 6b). Furthermore, through an electrochemical impedance calculation [64], the research group showed that the diffusion coefficient of Sodium-ion in the low-potential platform region was similar to that of graphite, confirming the intercalation mechanism of the platform region.

Ji [65] regulated the structural characteristics of HC by means of heteroatom-doping and revealed the storage mechanism of Sodium-ions in the material. A high temperature annealing treatment of HC resulted in a decrease in slope capacity. High temperature annealing would remove defect sites from the surface of the material, leading to a decrease in its sodium storage capacity. Therefore, the slope capacity was attributed to the adsorption of sodium at defect sites. 

Moreover, P-doping shows a high reversibility and high capacity of sodium intercalation, B-doping increases the number of defects in the carbon plane (in the absence of Lewis base, boron can only form three bonds, and its doping is more likely to occur in the carbon plane and form defect sites), and the doping of sulfur and phosphorus increase the layer spacing of the graphite microcrystalline region by means of space occupations simultaneously (shown in Figure 6c). XRD, transmission electron microscope (TEM), and energy dispersive X-ray (EDX) were applied to confirm the conjecture; P-doping and S-doping also increase the layer spacing and sodium storage capacity of the low potential platform, indicating that the corresponding mechanism is intercalation. The high defect density introduced by B increases the sodium storage capacity in the inclined region, proving that the mechanism of sodium storage in the slope region is the reversible adsorption of defect sites (shown in Figure 6d). It is worth noting that the experiment also found that the B-doping site and Sodium-ion have high binding energy, which will increase the irreversibility of adsorption, so it is necessary to avoid introducing high-energy defect sites into the structure [65].

#### 3.3.3. Intercalation-Pore Filling

**Figure 7:**
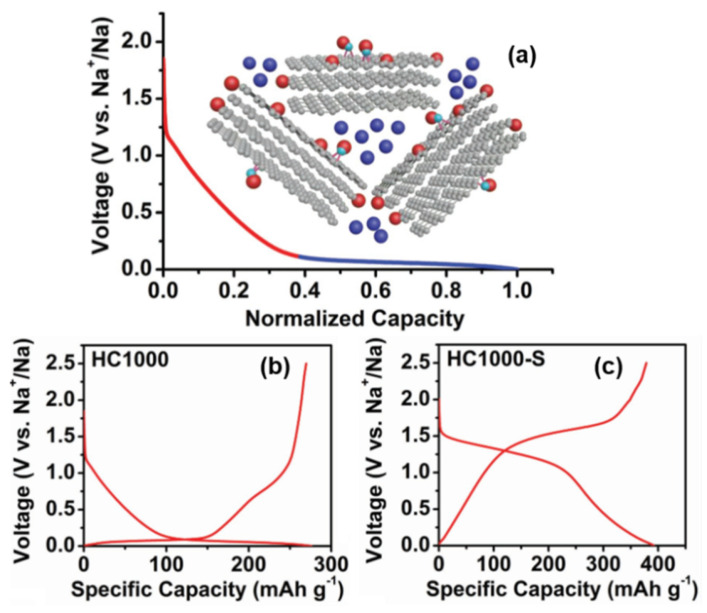
(**a**) Schematic illustration of the “adsorption-pore filling “mechanism for Sodium-ion storage in HC. (**b**) Galvanostatic charge and discharge curves in the 2nd cycle of 1000 °C pyrolytic HC. (**c**) Galvanostatic charge and discharge curves in the second cycle of 1000 °C pyrolytic HC–S composite.(in Na half-cells with 0.8M NaPF_6_ in DEGDME at a current density of 20 mA g^−1^) [66].

In addition to the two opposite mechanisms mentioned above, there are other sodium storage models in which there is no Sodium-ion intercalation between graphite layers in HC materials; instead, there is pore filling progress at the low potential plateau region, as shown in Figure 7a.

Xu [66] introduced an S element into the HC structure (HC–S composites were prepared by mixing and vacuum pyrolysis), and the same characteristic peak (002) of XRD indicated that sulfur did not enter the interlayer of graphite. There was no sulfur deposition on the surface of the carbon structure observed by high resolution transmission electron microscopy (HRTEM), so it was proved that sulfur was stably infused and dispersed in the microporous interval. In addition, it reduced the capacity of the platform area and provided strong evidence for the “pore filling” sodium storage mechanism of the low-potential plateau (as shown in Figure 7b,c). Their work also mentioned the correlation between the decrease in the surface defect concentration and the slope capacity, along with the effect of the electrolyte on the platform capacity; it was proven that there was no reversible Sodium-ion intercalation between the graphite sheet layers.

**Figure 8:**
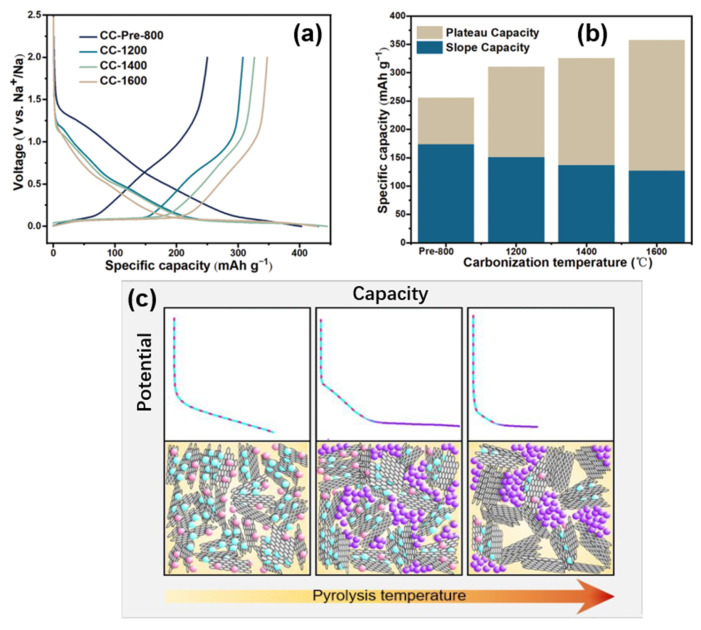
(**a**) Galvanostatic initial discharge and charge curves of cock-derived HC (prepared at different pyrolytic temperatures) at a current rate of 0.1 C (30 mA g^−1^). (**b**) Specific capacity of cock-derived HC from the different plateau (<0.15 V) and slope (>0.15 V) contributions (discharge capacity at the 2nd cycle) [67]. (**c**) Simple schematic illustration of the proposed sodium storage mechanism [68].

Hu’s group [67] also proposed a similar mechanism by adjusting the complex pore structure of a type of waste cork-derived HC. With the increase in pyrolysis temperature, one-dimensional-like morphology was formed in the pore structures, which promoted the ion transportation. The measurement results of the galvanostatic intermittent titration technique (GITT) showed that the lower the pyrolysis temperature, the lower the platform voltage and the higher the slope voltage, suggesting that the sodium storage potential is related to the microstructure of this HC (as shown in Figure 8a,b). As mentioned above, this work considers that there is no sodium intercalation process in the graphite microcrystalline region in the mechanism of sodium storage. Via mercury intrusion, nitrogen adsorption, SAXs, skeletal density techniques, various electrochemical analytical methods, and the in situ analysis of a well-designed pore structure, the correlation between the internal porous structure and the electrochemical properties of HC was obtained. Open-macropores are conducive to the stability of the carbon matrix; open-micropores have an influence on initial coulombic efficiency and closed-nanopores benefit the increase of the platform capacity; that is, they support the filling mechanism of the platform region. Besides, the filling process of sodium at this position is more complex than the deposition (such as forming metallic clusters).

Titirici’s group [68] prepared a glucose-based HC with different internal structures by temperature regulation; the sodium storage mechanism was modified based on the intercalation-adsorption model of Dahn. It was found that pore filling occurred in the low-potential plateau region, and the pores became more metallized with the increase in pore sizes, demonstrated by ex situ ^23^Na nuclear magnetic resonance (NMR) spectroscopy. As Figure 8c shows, the HC with various internal structures were prepared by adjusting the temperatures from 1000~1900 °C, the schematic diagram was made by linking the characterization of materials structure with the electrochemical properties. 

Relatively low pyrolysis temperatures have the highest defect concentration and the widest spacing between the sodium storage layers, although strong binding energy at some defect sites can lead to irreversible storage of sodium at these sites (also the first cycle of irreversible storage caused by the formation of an unstable solid electrolyte interphase, which is not discussed in this work). Due to the small pore area at this time, the pore filling amount is very small, resulting in an electric potential curve that is completely composed of slope capacity. With the increase in pyrolysis temperature, the crystallinity increases, some surface defects are removed, and the interval between the layers decreases slightly. However, the storage capacity of the pores increases with the increase in pore size, so the platform area capacity appears and the slope area capacity decreases. At higher pyrolysis temperatures (about 1700~1900 °C), the number of defects plunges, so the capacity of the slope area decreases significantly. In addition, with the increase in pore size, the diffusion pathway is closed, thus some pores cannot be used for sodium storage, and it is obvious that the platform capacity also decreases. The same hypothesis was also confirmed by X-ray and neutron scattering, ex situ ^23^Na NMR spectroscopy, electrochemical characterization, and Density Functional Theory (DFT) simulation.

#### 3.3.4. Adsorption-Intercalation-Pore Filling

**Figure 9:**
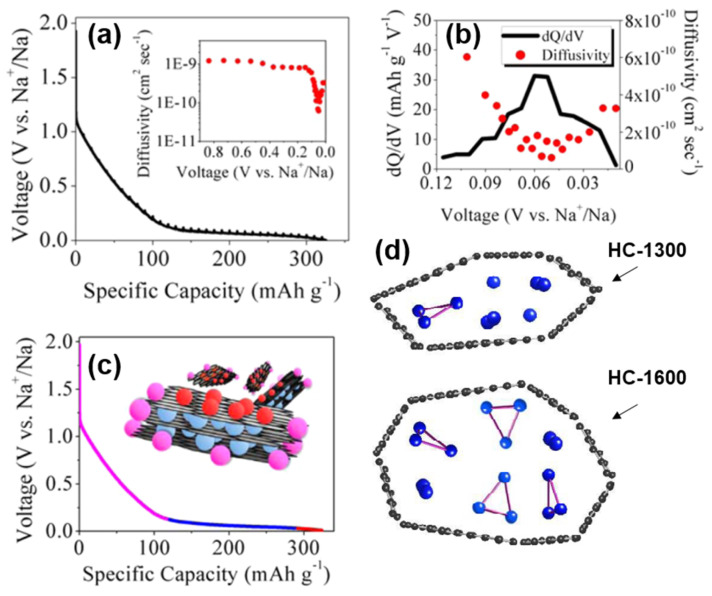
(**a**) GITT profile and diffusion coefficient as a function of states of charge (inset). (**b**) dQ/dV plot from 0.12 V to 0.01 V with corresponding diffusivity values within which a sudden small scale change can be observed. (**c**) Schematic of proposed Sodium-ion “adsorption-intercalation-pore filling” storage mechanism [69]. (**d**) Schematic models of sodium storage in HC nanopores of two different pyrolysis temperatures [71].

Ji’s group [69] adopted the adsorption-intercalation theory of Dahn, and also confirmed the defect adsorption behavior in the high-potential slope region, and the Sodium-ion intercalation behavior at the low platform region in the diffusion coefficient characterization experiment of sucrose-based HC. The diffusion coefficient of the high potential zone was significantly higher than that of the low potential zone, which was attributed to the fact that the surface defects of the graphite layer preferentially adsorbed Sodium-ions, and the barrier of Sodium-ion repulsive force that needed to be overcome during the intercalation process. More importantly, as shown in Figure 9a,b, the diffusion coefficient expands significantly around 50 mV, which was likely neglected in the previous characterization and calculation [70]. Therefore, a unique nanopores filling mechanism in the region of 0~0.05 V is proposed.

Subsequently, the state of sodium in HC was analyzed by the combination of NMR and nitrogen adsorption [71], and the storage model of quasi-metallic sodium clusters in nanopores was proposed in this paper (shown in Figure 9d). The pore size is limited and the storage of the Na_3_ clusters on the pore surface is prevented at insufficient pyrolysis temperatures (less than 1300 °C). However, at higher pyrolysis temperatures, the cost of such storage in nanopores is the decrease in surface defect concentration and a plunge in slope capacity, so such filling behavior may be minimal. The schematic diagram of the adsorption-intercalation-pore filling mechanism is shown in Figure 9c; the model has since been proven and is thought to better explain most of the results [72,73].

To sum up, the specific mechanism of sodium storage in the low-potential platform region of HC materials is still a debatable topic. The behavior of Sodium-ion insertion, pore adsorption and filling, as well as the deposition on the surfaces depends significantly on the complex HC microstructure. It requires more precise characterization and more specific structural regulation methods to reveal whether these behaviors occur simultaneously or independently. From the other aspect, the current mechanism of sodium storage in high-potential slope curves is the same, namely adsorption on material surfaces, edges, and defect sites. It is of great significance to explore the mechanism of sodium storage for directional synthesis of high-performance HC materials. As the work of Liu [63] showed, the adsorption-embedment theory was confirmed by adjusting the internal size of the material; increasing the capacity of the slope area requires maintaining a large defect density, but it will increase the irreversible influences of solid electrolyte interphase (SEI) film on ICE. Therefore, Sodium-ion intercalation with a stable voltage platform should be strengthened. Under the guidance of this understanding, a highly ordered layered structure with an appropriate layer spacing (0.37~0.38 nm) was designed in order to improve the storage capacity of sodium in the low potential platform region, which benefits the coulomb efficiency to a large extent. Eventually a non-porous HC material with a high reversible capacity and coulomb efficiency was developed and realized in practical application.

**Figure 10:**
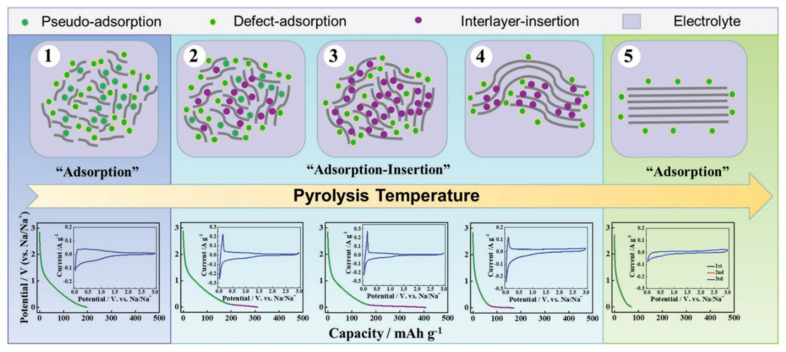
Schematic illustration of the extended “adsorption-intercalation” model [74].

In addition, while exploring the mechanism of sodium storage, methods for regulating the HC structure, and methods for characterizing the material properties were gradually completed. Xu’s group [74] established an extended “adsorption-intercalation” model, as shown in Figure 10, dividing the evolution of the structure and the storage of sodium into five stages and three behaviors. Disordered carbons at low pyrolysis temperatures (with a layer spacing larger than 0.4 nm) tend to exhibit “pseudo-adsorption” behavior on the material surface (such as adsorption at defects, edges, heteroatomic centre, etc.). Along with the temperature increasing, the disorder degree decreases, and the interval between layers is about 0.36~0.4 nm, which is enough for the Sodium-ions to achieve a reversible interlayer intercalation. At this time, the pseudo-adsorption behavior is still continuing, which is very similar to the traditional adsorption-intercalation theory. The enhanced rearrangement at high temperature leads to the deepening of the HC graphitization, and the layer spacing is below 0.36 nm, which makes it difficult to complete the insertion. The residual defects and pores on the surface attribute to the last slope capacity.

It should be noted that the process of storing and releasing Sodium-ions is dynamic, and it may be defective to judge the sodium storage behavior of electrodes based on the experience of electrochemical kinetics alone. More accurate and visible measurement methods are needed to prove the sodium storage mechanism of HC. In summary, it is necessary to have a more comprehensive and deeper understanding of the structure of HC to reveal the sodium storage mechanism of HC materials in SIBs, to guide the controllable synthesis of anode materials, and to approach the industrialization of high-efficiency battery materials.

## 4. Modification and Optimization Strategies of HC

The practical application of HC is limited by its low-rate capability, cycle stability and the low initial coulombic efficiency, especially. Due to the high specific surface area of HC, the contact between the electrolyte and electrode material surface will be strengthened, forming an SEI film, which will block the path of Sodium-ion transportation and lead to an irreversible capacity after the first cycle of charge and discharge. The effect of SEI films on the first cycle electrochemical properties of materials has been studied and reported as early as the 1990s [35,75]; the same problem exists in LIBs, where the decomposition and coating of the electrolyte on the surface of the material impedes the movement of electrons and ions, which is also directly related to the specific surface area of the material. Subsequently, Ji’s group [76] characterized the surface area and structure of the material by means of XRD and BET, proving that the increase of measurable porosity was closely related to the decrease of reversible capacity, and proposed that the carbon atoms contributed to the surface area and porosity and were “exposed carbon atoms”. The contribution of these atoms to Sodium-ion storage is very low, probably because the exposed carbon atoms become nucleation sites for SEI phase formation, forming a passivation layer on its surface and leading to an irreversible storage in the first cycle.

Cao [42] predicted through theoretical simulation of the experimental results that defects in the carbon layer capture Sodium-ions and generate a repulsion electric field. High defect sites and sodium binding energy will not only hinder the further entry of Sodium-ions, but also lead to the increase of the irreversible capacity in the first cycle. Therefore, in order to synthesize high-performance HC anode materials that can be put into practical applications, the modification and optimization strategies of HC materials are particularly important. Various optimization strategies reported in recent years are summarized as follows. 

### 4.1. Heteroatom Doping

Heteroatom doping can change the electron/ion state of the active material and favor Sodium-ion storage. For carbon-based anode materials, the main dopants are non-metallic atoms, such as B [65], N [77], F [78], P [79], S [80], and O [81]. The heteroatom doping can not only adjust the intrinsic structure of carbon materials, but also introduces a variety of different functions due to its own properties. For example, fluorine, because of its strong electronegativity, can weaken the repulsive force of Sodium-ion insertion, and thus lower the energy barrier of Sodium-ion insertion. Wang [78] prepared an F-doped HC derived from lotus petioles, which had a reversible capacity of 228 mAh g^−1^ and remained at 99.1% capacity retention for up to 200 cycles.

Early in 2015, Yu [82] prepared N-doped porous carbon fibers using polypyrrole as a carbon source, focusing on the effect of nitrogen function on the electrochemical performance of the carbon anode. In the porous structure of fibrosis, N atoms provided enough active sites and showed a high capacity of 296 mAh g^−1^ at 0.05 A g^−1^, however, its ICE reached only 46% because of the irreversible adsorption of defects and heteroatom sites along with the formation of SEI films. Recently, N-doped HC anode materials with low ICE and a high capacity were also reported. Ma [77] prepared a porous carbon material rich in N using bamboo leaves as a precursor. Yang [83] synthesized a structurally stable high nitrogen-doped carbon material by combining metal-organic framework (MOF) materials. The anode made from this nitrogen-doped carbon material showed a high capacity of 198.2 mAh g^−1^ at 5 A g^−1^, but its coulomb efficiency was still less than 60% in the first cycle. Carbon sheets with a high N-content were prepared with okara [39], exhibiting an ultrahigh rate capability after carbonization and subsequent exfoliation. It showed a capacity of 258.9 mAh g^−1^ in the first cycle and remained at 247.5 mAh g^−1^ after 50 cycles. Its ICE is much higher than that of other reported heteroatom doped materials [40,84,85]. Its excellent performance was attributed to its special structures. However, the underlying causes need to be studied more deeply. In short, the synergistic effect of N-doping and defects in the structure [86] can increase the sodium storage performance of HC materials, and conversely, improve the influence of SEI film, leading to the inevitable low ICE, which is the main problem to be overcome.

**Figure 11:**
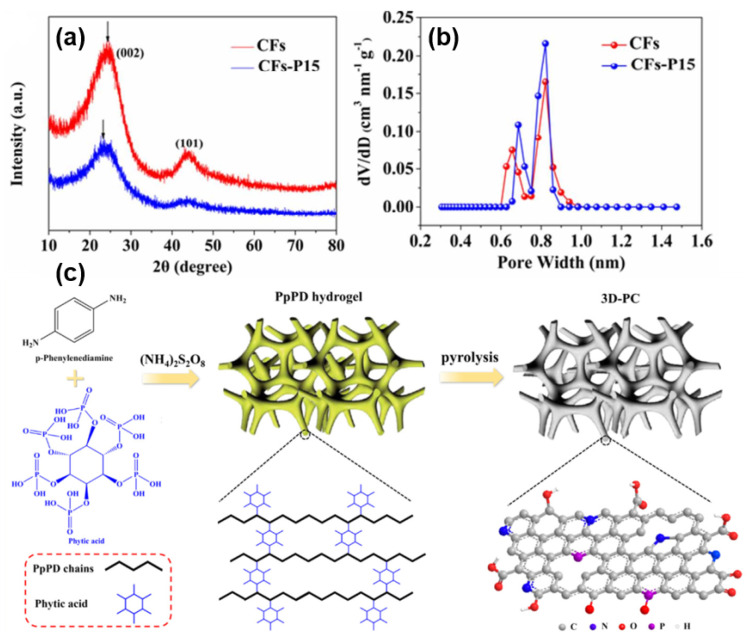
(**a**) XRD patterns. (**b**) The pore size distributions of carbon samples with or without P-doping [88]. (**c**) Schematic illustration of the design with multi-atomic doping and three-dimensional-porous structure [95].

Ji [79] first explored the influence of P-doping on the sodium storage performance of SIB anode material, and prepared P-doped carbon nanosheets with an excellent rate performance. Compared with N, P-doping can not only provide electrochemical active sites, but also increase the layer space to facilitate the reversible intercalation of Sodium-ions and change the electronic states of the carbon sheets to promote ion adsorption in the electrolyte. Using three-dimensional well-ordered porous phosphorus doped carbon as an anode for sodium storage was also studied [87], and a theoretical calculation further confirmed that P-doping can increase the interlayer distance and adsorption capacity of Sodium-ion, reaching the capacity of 270 mAh g^−1^ at 0.2 A g^−1^ after 800 cycles and a remarkable rate capability of 140 mAh g^−1^ at 10 A g^−1^. Wu [88] prepared HC nanofibers via electrostatic spinning and simple heat treatment. By comparison (as shown in Figure 11a,b), an excellent performance of the P-doping was demonstrated, and it was confirmed that a P–C bond can increase the electron density near the Fermi-level, thus enhancing the electronic conductivity of the material. Similarly, Jiang’s Group [80] prepared S-doped nanoscale carbon fibers using an industrial waste bacterial cellulose as a carbon source. With the synergistic effect of the carbon structure, S-doping and defect sites, a larger layer spacing, and the electrochemical activity of the S–C bond play significant roles, still providing a capacity of 310 mAh g^−1^ after 1100 cycles at 1 A g^−1^. It is considered that S atoms are doped in the interlayer of carbon [89]. Hong and Zhang [90] studied the influence of S-doping on the layer spacing in two kinds of HC with a defect free structure and a defect structure by the first-principles method, and prepared an S-doped HC with a low porosity and larger layer spacing. The reversible capacity of the material remained nearly 200 mAh g^−1^ after 4000 cycles at 1 A g^−1^, and its ICE and cycling performance were significantly higher than those of the materials mentioned above. Phosphate produces electron holes near the Fermi level, which is often taken as evidence that the p-type semiconductors can improve electronic conductivity. Recently, an in-depth study [91] of the charge distribution has shown that electron holes are mainly generated by the phosphate doped in HC with a higher reducibility than the surrounding carbon atoms. This finding further explains the basic principle of enhancing the electrochemical performance of P-doped HC. 

In addition to the typical single-atom doping examples mentioned above, there are also many reports on double-doping and tri-atom co-doping in recent years. Wang [92] synthesized the layered carbon material co-doped with N and S by small molecule dithiooxamide using a facile self-templating strategy. Combined with the synergic characteristics of N and S doping, defect sites are formed in the layer to increase the storage capacity of sodium while expanding the layer spacing. Simultaneously, the two heteroatoms improve the electron and ion transfer rate of the material, providing an excellent performance of 213.6 mAh g^−1^ at 1 A g^−1^ for up to 2000 cycles. In order to improve the low carbon yield from starch direct pyrolysis, Huang’s Group [93] prepared an N and P co-doped porous carbon using ammonium polyphosphate (APP) as both the dopant and crosslinking agent. DFT calculations based on first-principles confirmed that the co-doping of the N and P atoms can improve the conductivity of carbon materials and the adsorption capacity of the Sodium-ions, as well as reducing the diffusion barrier of Sodium-ions between graphite layers. The research team also used a high-yield and low-cost biomass insect feces as a precursor system to prepare an O, N, P, and S co-doped porous carbon material, which achieved an excellent cycling performance of capacity retention above 95% at 1000 cycles [94]. In contrast, Song’s Group [95] prepared three-dimensional porous carbon materials with N, O and P co-doping by simple pyrolysis of the poly(P-phenylenediamine) (PpPD) hydrogel with phytic acid as a dopant and crosslinking agent. The mesoporous structures formed by the three-dimensional PpPD hydrogel network gives the anode material abundant defect sites and ion transport paths, and it serves a better electrochemical performance of 332 mAh g^−1^ at 0.05 A g^−1^, 139 mAh g^−1^ at 10 A g^−1^ and a wonderful cycling stability of 98.9% retention after 1000 cycles at 5 A g^−1^; this design is shown in Figure 11c.

In summary, different heteroatom doping has similar effects; for example, sulfur, phosphorus, and fluorine doping can improve layer spacing and facilitate the diffusion of Sodium-ions; boron and nitrogen can create more defect sites and increase the adsorption capacity; and nitrogen, oxygen and phosphorus doping can increase the electronic conductivity of materials [81,96,97]. Heteroatom doping will also bring disadvantages, such as low ICE caused by a larger specific surface area in doping HC, and the hidden danger of structural deformation caused by heteroatoms, such as S, P, F in the charge and discharge process, as well as irreversible adsorption caused by the high binding energy of B [98]. In this view, controllable heteroatom doping is a key step for the specific properties of materials. Therefore, further attention should be paid to the specific sites of heteroatom doping and the reasons for its influence on the structure, as well as the reasons for the influence of the introduction of heteroatoms on electrical properties. Therefore, multi-atom co-doping arises at the right moment. In recent years, the electrochemical performance of anode materials reported by co-doping is excellent, which benefits from the synergistic effect of various atomic properties. In addition, various efforts have been made to find low-cost heteroatom-doped HC preparation methods [39,40,94]. For example, the biomass carbon source itself is rich in a variety of heteroatoms, which will self-dope into the HC structure during the carbonization process to provide excellent performance, but its synthesis has certain uncontrollability [39,77,78]. In future works, the specific principles of atomic doping should be the focus of further investigations. Understanding the mechanism and optimizing the synthesis process can provide practical significance for the industrial development of anode materials.

### 4.2. Structure and Morphology Designing

Heteroatom doping can change the microstructure of the material. In addition, the structure and morphology can also be designed by other means. Sodium-ion has a larger ionic radius and slower kinetic properties than lithium-ion, so increasing the diffusion channels and shortening the diffusion path will effectively improve the rate performance of the electrode. It is reported that various kinds of morphology designs can serve as anode materials, such as a zero-dimensional carbon dot [99], one-dimensional carbon fiber [88] and carbon tube [49], two-dimensional carbon sheets [79], three-dimensional carbon microspheres [100] and carbon framework [101], etc. Different dimensions of morphology designing will obtain different structural characteristics, which exhibit a hollow structure, porous structure, layered structure, and other different morphology. Many also combined with heteroatom-doping to improve capacity performance and to obtain excellent electrode materials.

**Figure 12:**
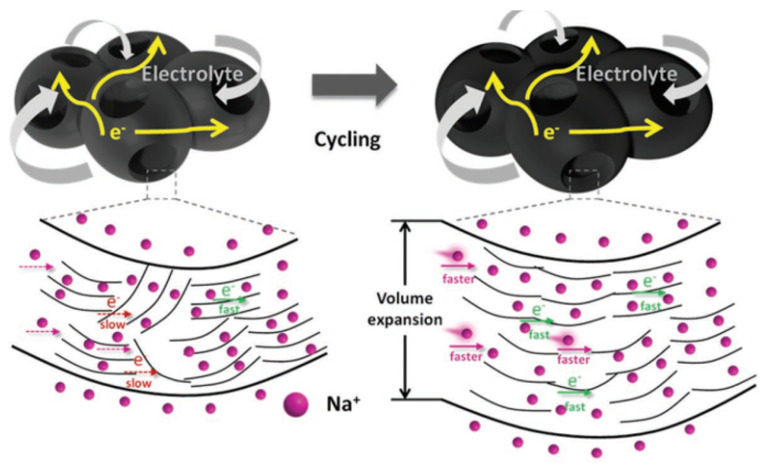
Schematic illustration of the heteroatom-doped mesoporous hollow carbon spheres, whose structure can also maintain stable and expanded Sodium-ion transport after volume expansion [103].

Porous carbon microspheres, including ordinary nitrogen-doped carbon microspheres (N–CS) and porous nitrogen-doped carbon microspheres (P–N–CS), were prepared by the template method [102]. The carbon microspheres with larger pore size and higher pore density could be prepared by pore-forming. Combined with the higher surface area and N-doped performance, the carbon microspheres exhibited a capacity of 155 mAh g^−1^ at 1A g^−1^ and almost no capacity change after 600 cycles. In recent years, the morphologies of HC materials have been designed in a variety of ways. In 2019, Sun [103] prepared mesoporous hollow carbon spheres. This unique structure (as shown in Figure 12) provides a large electrode/electrolyte interface that facilitates electrolyte diffusion, increases electron and Sodium-ion transport rates, and buffers the volume expansion of the electrode material during the cycle. With S and N co-doping, the materials show an extremely high rate capability of 144 mAh g^−1^ at 20 A g^−1^ and even rises to 180 mAh g^−1^ after 7000 cycles, which is attributed to structural changes such as the recombination of the carbon sphere structure and the increasing distance of the carbon layer during the charging and discharging process. Lu [104] prepared uniformly concentrated and distributed ultra-porous carbon nanosheets with rich carbonyl and hydroxyl active groups on the surface. Such oxygen doping not only provided a high conductivity and sodium storage capacity [81], but also provided a new reaction potential of a high voltage plateau region (0.4~0.7 V) and an excellent rate performance of 145 mAh g^−1^ at 5 A g^−1^ with a favorable cycling stability.

**Figure 13:**
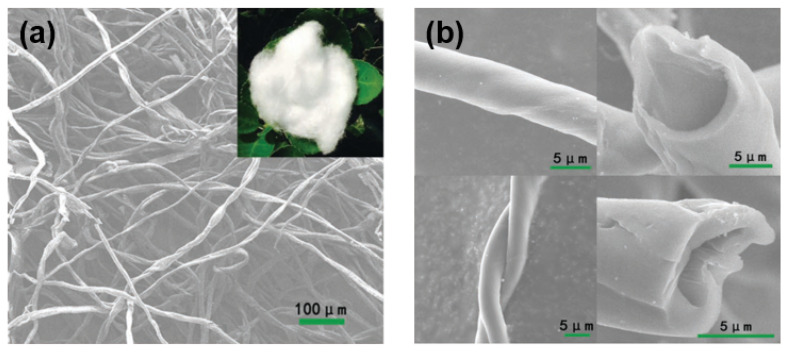
(**a**) SEM image of carbon source cotton. (**b**) The magnified SEM images of the carbonized cotton with a clear and visible hollow structure [49].

As shown in Figure 13, one-dimensional materials and HC microtubules, were reported earlier by Hu’s group [49]. The unique hollow tubular structure gives the anode material high capacity and rate properties. During the test of a full battery composed of a copper, iron and manganese metal oxide cathode, a high reversible specific capacity of 290 mAh g^−1^, an average operating voltage of 3.2 V, and an energy density of about 207 Wh kg^−1^ were obtained. For one-dimensional materials, excellent work has been performed [101], where a large number of carbon quantum dots (CQDs) were easily synthesized using acetone, and these monodisperse CQDs self-assembled to form carbon frames in a high-temperature argon atmosphere, which provides more sodium storage sites and improves the infiltration area of electrolyte, and reduces the diffusion length of the Sodium-ions. This anode material can reach a capacity of 90 mAh g^−1^ at 20 A g^−1^, and the capacity increases from 130 mAh g^−1^ to 137 mAh g^−1^ after 5000 cycles at 5 A g^−1^.

Furthermore, Yuan [105] synthesized flower-like nitrogen-doped hierarchical porous carbon networks (NHPCN) by using the self-template method and a single precursor. It has a high-level spaced graphite layer, ultra-thin two-dimensional nanostructure, and a porous three-dimensional network. With the help of this flower-like structure, the sodium storage properties and kinetic properties are greatly improved, and its cycling stability and rate performance are also very high due to the stability and high solution wettability of the structure. It shows 453.7 mAh g^−1^ at 0.1 A g^−1^ and 242.5 mAh g^−1^ at 1 A g^−1^ with almost no capacity loss after 2500 cycles, which is much higher than the normal N-doping strategy of ordinary anode materials. Interface engineering is also an important method of morphology control to solve the problem of insufficient ICE in HC materials. The FeS_2_ nanoclusters were uniformly inserted into the N and S co-doped carbon matrix, and the Fe was bound to the active sites of N and S on the carbon layer, this defect-repairing site will facilitate the decomposition of SEI film formed by electrolyte on the surface, and it will also form a two-dimensional SEI film itself as a crystal nucleus to increase the conductivity of the material surface [106]. The interfacial modification and heteroatom doped anode material obtained in this paper has an amazing reversible capacity of 749.6 mAh g^−1^ at 0.1 A g^−1^ and 401.9 mAh g^−1^ at 10 A g^−1^, with almost no capacity change at 3 A g^−1^ after 5500 cycles. Although this interface engineering process is limited by the choice of electrolyte, it provides an effective approach to reduce the influence of SEI film on ICE.

In summary, material structure undoubtedly has an important influence on the properties of materials. For HC materials, the carbonization temperature, precursor type, heteroatom doping, interface modification and other factors will affect their morphology characteristics. It is worth noting that some intrinsic morphology of biomass carbon sources has been used to synthesize materials with excellent properties. Hollow kapok fibers were used as a carbon source to synthesize one-dimensional micronanotubes [107] with excellent low surface area and interfacial transport properties, a reversible specific capacity of 290 mAh g^−1^ at 0.1 A g^−1^ and ICE up to about 80%. Tian [108] synthesized the two-dimensional carbon nanoplate from natural mushroom spores rich in chitin and cellulose, and formed the whole battery with Na_3_V_2_(PO_4_)_3_, exhibiting a high power density of 199.2 Wh kg^−1^, along with the advantages of industrialization due to the low cost. Cao’s group [109] synthesized one-dimensional HC fiber materials using low-cost waste paper as a carbon source, and adjusted its structure and properties with the change of pyrolysis temperature, achieving a capacity of up to 319.6 mAh g^−1^ and a high cycling stability of 99.3% capacity retention rate after 100 cycles. Similar to heteroatom doping, the regulation of the structure has advantages and disadvantages. Although the porous structure and layered structure are helpful to the diffusion dynamics of Sodium-ions, they also bring a high specific surface area and a high specific volume capacity, which have a certain influence on the energy density of the whole battery. To sum up, finding a safe, low-cost, and simple morphology regulation method is a focus of current application research, and it is necessary to have a certain grasp of the degree and mechanism of the regulation. The realization of fully controllable morphology regulation is of great significance to the industrialization of high-performance anode materials.

### 4.3. Preparation of Composite Materials

**Figure 14:**
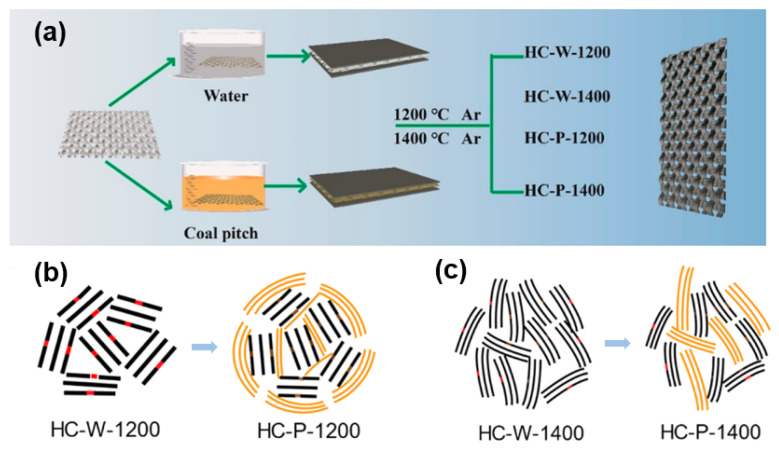
(**a**) Schematic diagram of the synthesis of different samples, HC–P in coal-pitch impregnation and HC–W in water impregnation. (**b**,**c**) Schematic diagram of structure: red dots represent the defect sites and yellow lines represent the product of coal-pitch (soft carbon coating) [112].

Heteroatom doping and morphology regulation can improve and modify the intrinsic properties of HC. In recent years, attention has been focused on developing carbon composite materials to enhance performance. From the selection of materials, carbon materials with different structures, such as soft and HC composites can achieve a cooperative storage of sodium. In early years, Hu and co-workers combined a low-cost soft carbon source pitch with a lignin-based HC material to prepare a composite anode that provides a cost-effective and performance-based synthesis strategy [110]. Later in 2019, inspired by Hu’s work, Titirici [111] prepared soft and HC composites using mesophase pitch (MP) as a soft carbon precursor and filter paper as a HC source. Compared with the pure HC materials or pure soft carbon materials, this composite material achieves a higher capacity and ICE. The high ordered structure and low defect of soft carbon can effectively reduce the irreversible sodium storage in the first cycle caused by the high specific surface of HC due to the formation of SEI film and the irreversible insertion of sodium-ions. At the same time, the high interlayer spacing of the HC material at the low carbonization temperature (1000 °C) provides sufficient capacity and binding sites. It shows 282 mAh g^−1^ at 0.03 A g^−1^ and 80% ICE with cost advantages. Subsequently, the research group of Wu and Chou [112] also adopted the method of soft carbon coating to solve the ICE problem of HC materials. A coating on the surface of an independent HC electrode obstructs the formation of defect sites and highly active oxygen-containing functional groups, and the coating is achieved through a pre-immersion strategy on paper towels (as shown in Figure 14a). After coating and annealing at 1200 °C, the HC material achieves 94.1% ICE and 99% capacity retention after 100 cycles at 0.02 A g^−1^. The formation of material surface defect sites and oxygen-containing functional groups are inhibited by coal tar pitch carbonization products, the hole is also blocked to reduce electrolyte decomposition, as is shown in Figure 14b-c, reducing the irreversible capture of Sodium-ions on the HC surface, thus high ICE and cycle stability are obtained.

**Figure 15:**
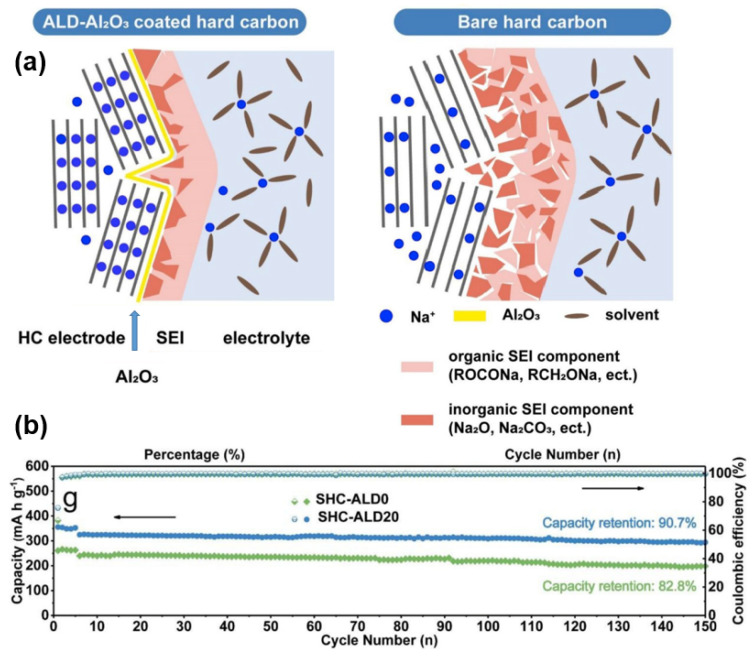
(**a)** Schematic diagram of the influence of an ALD–Al_2_O_3_ coating on the surface of SEI on HC. (**b**) Cycling performance of HC with or without an ALD–Al_2_O_3_ coating at a current rate of 50 mA g^−1^ (the current rate is 20 mA g^−1^ in the initial five cycles) [114].

As for other carbon materials, Lai’s group [113] directed carbon nanofibers to vertically penetrate through graphene sheets, constructing a robust carbon nanofiber interpenetrated graphene architecture. The carbon fiber inhibits the stacking of the graphene layers, and the three-dimensional structure provides fast sodium transport paths, providing excellent mechanical properties and electrical conductivity as an anode material. The authors also grew molybdenum disulfide in situ on the surface of the frame. This layered transition metal sulfide shows a different mechanism to what was discussed in this review of HC materials, so it will not be described in detail. However, the composite material prepared by this method can enlighten and guide the modification and development of HC anodes. In the same way, Cao [114] used atomic layer deposition (ALD) to coat Al_2_O_3_ film on the surface of HC as an “artificial solid electrolyte interface”. This artificial SEI film limits the decomposition of the electrolyte on the surface of the material, effectively improving the cycle stability of HC materials (as shown in Figure 15a). After 150 cycles of charge and discharge, the capacity retention rate of the coated anode material increases from 82.8% to 90.7%; the change of cycling stability is shown in Figure 15b. Al_2_O_3_ coating restrains the penetration of electrolyte and reduces the interfacial resistance of HC, so that more Sodium-ions are inserted into the graphite layer. Cao’s group put forward the critical thickness of this coating at about 2 nm through calculation and experiment, and the material maintains excellent electron and ion transport function under this condition. After coating, the reversible capacity increased from 260.9 mAh g^−1^ to 355 mAh g^−1^, and ICE increased from 67% to 75%. Du [115] modified MoO_2_ particles on an agaric-like carbon matrix to form a high-performance composite anode for SIBs. Different from the Al_2_O_3_ coating just mentioned, an MoO_2_ is coated on the carbon-based surface as an active material involved in the electrode reaction. Benefiting from its structural stability and high conductivity, it has a main sodium storage capacity of 193.5 mAh g^−1^ in 5 A g^−1^. The preparation strategy of this composite material also provides a new idea to explore the application of HC materials in SIBs.

**Figure 16:**
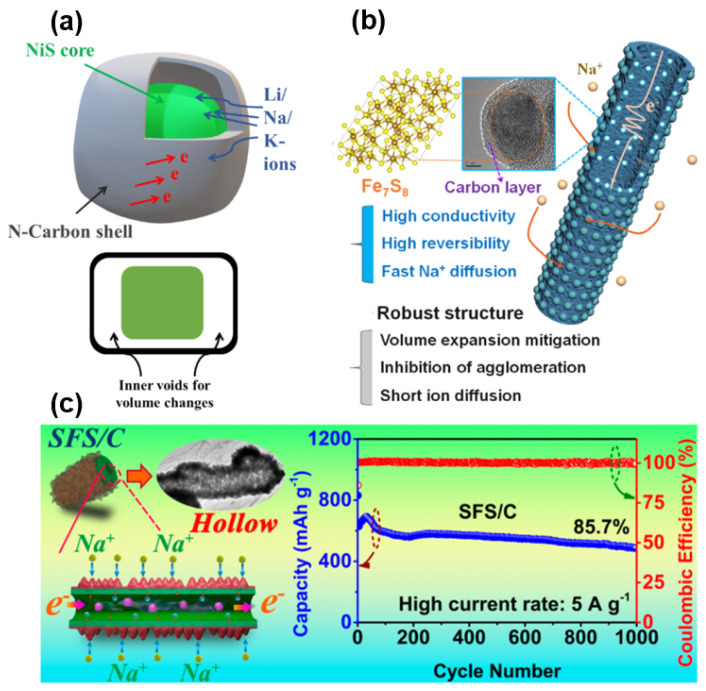
(**a**) Schematic diagram of the box-like core/shell NiS@Carbon that can transport and store Li/Na/K-ions [117]. (**b**) Schematic diagram of the NHCFs/Fe_7_S_8_ and the advantages of this material [119]. (**c**) Schematic structure of SFS/C (Sb_2_S_3_@FeS_2/_N-doped graphene) on the left, and the cycling performance on the right [120].

As for the transition metal sulfides and oxides mentioned above, this conversion-type sodium storage mechanism shows a high sodium storage capacity, but it is limited by a low conductivity and volume change during the reaction process. The HC materials reviewed in this paper have a different intercalated sodium storage mechanism. They also exhibit high electron and ion transport efficiencies and stable mechanical properties, so they can be used to compensate for the defects of the former. Compared with the other metal sulfide system, NiS shows a high electronic conductivity and theoretical specific capacity [116]. The groups of Yang and Zhang [117] collaborated to prepare NiS and nitrogen-doped carbon composites by using a strategy of polydopamine coating and post-processing, which has a unique boxed structure (as shown in Figure 16a). NiS is encapsulated in a carbon cage with Ni–N bonds, providing an ultra-high sodium storage capacity of 632 mAh g^−1^ at 5 A g^−1^ over 2000 cycles while maintaining high electron and ion conduction rates. Wu and Fan’s group [118] adopted Ni–MOF as the precursor to prepare Ni_3_S_2_ composite materials encapsulated in an N-doped porous carbon. The ultra-small size of the Ni_3_S_2_ itself reduces the stress of the material and shortens the Sodium-ion transmission path. The high-performance anode material for SIB was obtained by combining the high conductivity and stable structure of nitrogen-doped carbon material and the sodium storage behavior of the N-doping active site. Liu [119] embedded iron sulfide in a hollow N-doped carbon nanofiber NHCFs (as shown in Figure 16b). Fe_7_S_8_ not only has advantages in cost and resources, but also has good electrochemical activity. As the anode material of SIB, Fe_7_S_8_/NHCFs has a superb capacity and cycling stability of 517 mAh g^−1^ at 2 A g^−1^ after 1000 cycles and a considerable rate capability of 444 mAh g^−1^ at 20 A g^−1^. Luo and co-workers also modified bimetallic sulfide on the surface of hollow carbon nanorods using a two-part solvent method to prepare composite anodes [120]. Its electrochemical activity was attributed to the active carbon sulfide materials Sb_2_S_3_ and FeS_2_ coated with a carbon substrate, which accelerated electron/ion transportation in the carbon matrix. It also effectively alleviates the volume expansion in the long cycle and has excellent cycling stability, as shown in Figure 16c. Similarly, Sun [121] constructed layered hollow microflower-like heterojunctions, which encapsulated ZnS and CuS within the polydopamine-derived carbon framework. After 700 cycles, the capacity retention rate was nearly 100% and the rate capability of 341 mAh g^−1^ in 5 A g^−1^ was obtained. It should be noted that these carbon composite materials, made by coating carbon materials onto transition metal sulfide (e.g., forming a microboxed structure [122], layered nanobox structure [123] and a core-shell structure [124]), have a very different main mechanism of sodium storage and electrode reaction active substances from other HC materials mentioned in this review. What is the same is that the researchers have found and utilized the advantages of carbon as an anode material for improving the electrical conductivity and mechanical properties of the materials.

A carbon matrix and carbon coating were utilized to prepare a conversion-type composite anode and showed considerable storage capacity properties, rate capability and cycling stability. The preparation process, industrialization, cost, and resource cannot be ignored. Taking these into account, the modification strategy of hard and soft carbon composite has great advantages and commercial prospects. It is undeniable that other composite strategies also have guiding significance for the utilization and modification of HC materials. The controllable preparation of facile, low-cost, and high-performance composite anode materials is the main exploration direction nowadays.

### 4.4. Optimization of Battery Conditions

In addition to the design and regulation of the preparation process, a battery’s performance is also affected by many other conditions. The anode of SIBs can be modified by means of pre-treatment, adding additives, and electrolyte optimization. 

#### 4.4.1. Electrolyte Optimization

The irreversible capacity loss of an HC anode has an inseparable correlation with electrolyte [125], so the optimization of electrolyte conditions in the battery is of great importance. Early in 2012, Ponrouch [126] investigated the performance benchmarks of different sodium salts and various electrolytes, and systematically studied the optimization of electrolyte viscosity, conductivity, electrochemical properties and thermodynamic stability, establishing an excellent standard electrolyte system of NaPF_6_ in EC:PC. Theoretical simulation has also been applied to optimize the conditions of SIB electrolytes [127]. In addition to the thermodynamic factors, such as solvation energy, it has also been proven by the method of DFT that the binary solvents of EC: PC are the most favorable electrolyte systems. It is instructive that the simulation-experiment method is very suitable for assisting in the selection of appropriate full battery electrolyte conditions.

However, as has been mentioned many times before, HC materials suffer from unstable decomposition in carbonate electrolyte, common ester electrolytes such as ethylene carbonate (EC), propylene carbonate (PC) and in methyl carbonate (EMC). The coulomb efficiency can only be maintained at 10–40% in the first cycle. Schafzahl [128] reported a new system of dimethoxyethane (DME) with sodium bis(fluorosulfonyl)imide (NaFSI) electrolyte in SIBs. The advantages of NaFSI and DME are combined to form a stable SEI film, which is found to be composed of an organic ether and salt decomposition products through the characterization of EDX, Fourier transform infrared spectroscopy (FTIR) and NMR. Since then, there have been many reports on electrolyte optimization in SIBs, and it is confirmed that the loss of sodium storage capacity of HC in common ester-based electrolytes is reduced in ether-based electrolytes [129]. Yang’s Group [130] observed the formation of thinner, more stable and more compact SEI films with ionic conductivity in ether-based diglyme electrolyte, and confirmed that ether solvent could promote the diffusion of electrons and ions on the surface of carbon materials with a high specific surface area, and could change the formation dynamics of SEI films. Herein, rGO with a high specific surface area was selected as the carrier for the performance comparison between ester and ether solvents, and the coulomb efficiency difference in the first cycle could be nearly doubled. Although HC materials were not chosen for testing, the results of this comparative test have significant implications for the optimization strategy of electrolytes with an HC anode and open up a general SEI-modifying strategy that is not limited to carbon microstructure.

#### 4.4.2. Anode Pre-Treatment

**Figure 17:**
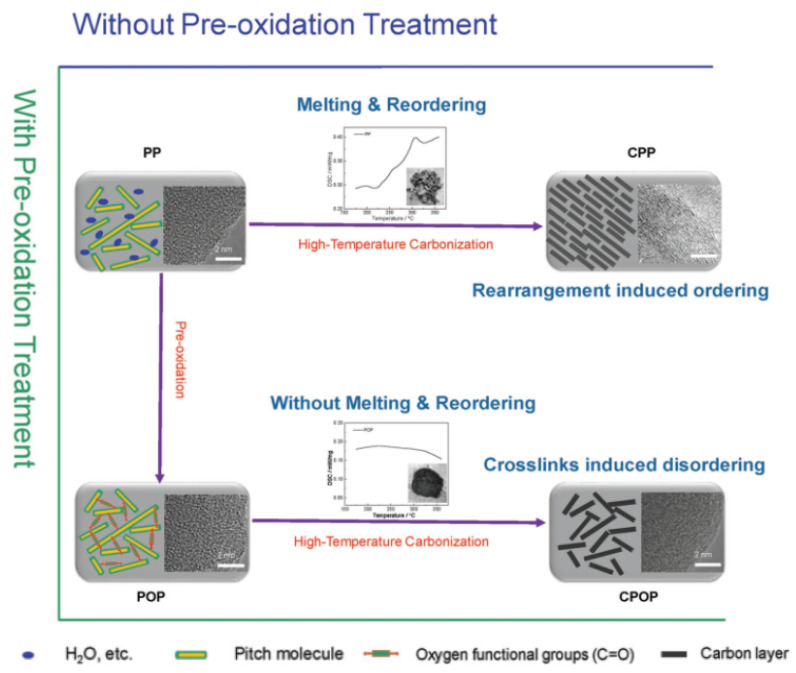
Schematic illustration of the carbon with or without the pre-oxidation treatment, including DSC curves and SEM images with the schematic diagram [26].

Another important means to optimize battery conditions is pre-treatment. Pre-oxidation can improve the electrochemical properties of HC materials from the perspective of electrode materials. Hu pioneered a simple pre-treatment method that could be extended to other precursors [26] through pre-oxidation. Crosslinking in a low temperature oxidation process was ensured, while rearrangement in a high temperature carbonization process was inhibited, maintaining a high degree of disorder in HC (shown in Figure 17). The reversible capacity increased from 94.0 mAh g^−1^ to 300.6 mAh g^−1^, and the ICE rose up to 88.6% from 64.2%. Recently, it has been proven that HC materials with expanded carbon interlayers could be made by the controlled introduction of oxygen-containing functional groups [131]. This pre-treatment greatly improved the capacity and performance of the raw commercial HC from 270 mAh g^−1^ to 341 mAh g^−1^ in 20 mA g^−1^. Kinetic measurement and theoretical calculation results show that the introduction of oxygen-containing functional groups expands the distance between the carbon layers, promotes the diffusion of sodium-ions, and enhances the adsorption capacity of sodium-ions. On the other hand, Wang [132] considered that the influence of unremoved heteroatoms and defects on HC lacks an experimental confirmation. Therefore, they used a pre-treatment method in which a mixture of organic vapor and argon is introduced during the annealing process of the precursor, to precisely regulate the heteroatoms and defects in the HC layer by controlling the atmosphere. If the vapor of cyclohexane is introduced, for example, the decomposition products react with the oxygen-containing functional groups on the surface and fill the defects of the carbon layer. Following this engineering treatment, this kind of HC anode exhibited a high ICE up to 85%, and the full-cell consists of HC//NVPF with an energy density of 239 Wh kg^−1^.

Pre-lithium is a pre-treatment method that significantly improves lithium capacity in LIBs. Balbuena and Li [133] applied pre-lithium to SIBs by ball milling Si with Li powder and contacting them with a tetraglyme-based electrolyte. It formed a SEI film via a reaction with the active substances, hindering the transfer of Sodium-ions and reducing the open-circuit voltage. This operation avoids the danger and instability of Na powder. Meanwhile, the lithium replenishment strategy in LIBs is adopted and the ICE in the HC anode of SIB is improved. The pre-treated HC anode has a specific capacity of about 150 mAh g^−1^ in 1 A g^−1^ and an ICE greater than 92%. This method provides practical significance by combining theory with experiment. However, the process is complex and resource problems still exist, so an independent “pre-sodiation” strategy belonging to SIBs still needs to be explored.

**Figure 18:**
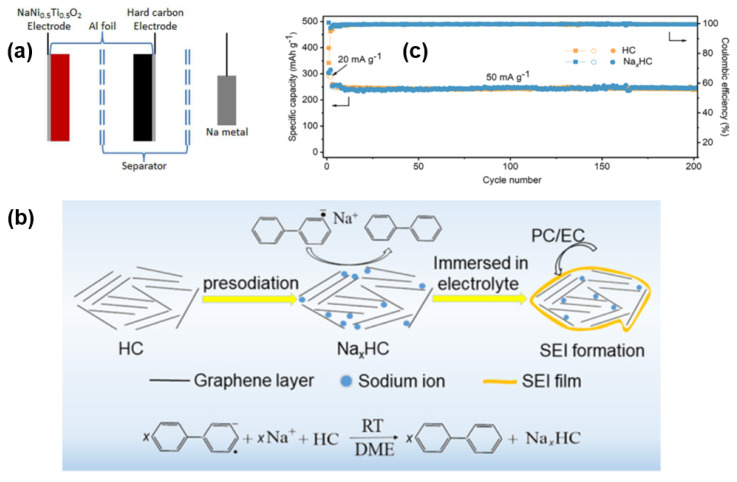
(**a**) Schematic illustration of three-electrode SIB, which use Na foil as the reference electrode [134]. (**b**) Schematic diagram of chemical pre-sodiation progress. (**c**) Cycling stability of HC and Na_x_HC electrodes at 0.05A g^−1^ [135].

In 2012, Ai and Lai jointly constructed a new pre-sodiation method [134] using a three-electrode method. A sodium foil was introduced as a reference electrode to complete the sodiation of the HC anodes (as shown in Figure 18a), and the ICE was increased to 73%. It provides the feasibility to achieve the “pre-sodiation” of SIBs in spite of the difficulty of controlling the unstable sodium metal electrode, the complexity of the method and the difficulty in achieving industrialization. Qian’s Group [135] has proposed a method of chemical pre-sodiation using a sodium diphenyl reagent to achieve a mild and efficient sodium supplement, which makes up for the problem of irreversible capacity loss. Combined with an Na_3_V_2_(PO_4_)_3_ cathode to construct a full battery, it shows a high ICE of 95.0% and an energy density up to 218 Wh kg^−1^. As shown in Figure 18b, the pre-sodiation depth can be precisely controlled by adjusting the pre-treatment time by immerging the HC electrode in an Na-bp solution due to the strong sodiation ability of Na-bp. Xu [136] pioneered a “foreign SEI” pre-treatment strategy in which a complete SEI film was formed by circulating in an ester electrolyte in advance, and then the HC electrode was stabilized in the ether electrolyte. By combining the stability of an SEI film formed by the decomposition of the ester electrolyte with the fast transport performance of the Sodium-ion in an ether-based electrolyte, an HC anode material with high cycling stability and rate performance was obtained through this synergistic action. It retained 200 mAh g^−1^ in 0.5 A g^−1^ after 1000 cycles. Furthermore, to compensate for the sodium irreversible capacity, a sodium-rich cathode can be used to optimize battery performance [137]. Such a cathode containing sodium can provide Sodium-ions to alleviate the irreversible sodium loss during cycling, thus improving the energy density of the full battery. It is also possible to use a sodium-supplement reagent (cathode sacrificial additive) that is oxidized as a cathode in the cycling to provide the sodium-ion capacity [138]. Obviously, both methods rely on metallic sodium or its active compounds (Na_3_N or Na_3_P), which inevitably leads to safety problems. Therefore, their commercial potential is bound to be limited by safety risks, and a more stable sodium supplement strategy needs to be explored.

#### 4.4.3. Self-Supporting Anode

**Figure 19:**
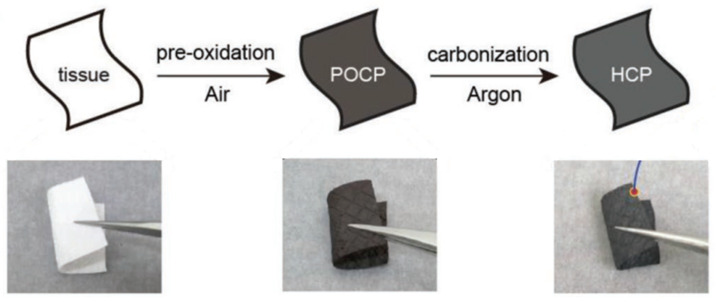
Schematic illustration of the preparation procedure for HC paper (HCP), and the digital photos of tissue show their flexible and self-supporting characteristics [142].

In the preparation of a full battery, the addition of a binder has a certain influence on the performance of the conductive carbon anode; for example, volume expansion in the process of charge and discharge will lead to a bad contact between the electrode and collector. In order to alleviate the inhibition of ionic diffusion and electron transfer by binders, self-supporting anodes have been studied continuously in LIBs [139]. Similarly, in recent years, self-supported anodes in SIBs without binders have been developed [140]. Yu [141] reported a simple preparation strategy for self-supporting HC anodes by constructing three-dimensional interconnected carbon nanofiber films by carbonizing bacterial cellulose, which can be directly used as an anode for SIBs. This synthesis strategy has the advantages of easy amplification, resource friendliness and a simple preparation process, and provides a new idea for optimizing HC anode materials. Wu [142] designed a self-supporting HC paper (HCP) derived from tissue by simple pre-oxidation and carbonization (as shown in Figure 19) to obtain up to 91.2% ICE and a great cycling stability in ether-based electrolyte. The low cost and controllability of this strategy make it commercially promising. Similarly, Xu’s Group [143] prepared a nitrogen-rich porous flexible carbon film as a free-standing anode by using inexhaustible chitosan as a carbon source, and good energy storage properties of 236 mAh g^−1^ in 2 A g^−1^ after 70 cycles has been realized. It can also be used as an excellent anode material for a potassium-ion battery. The design of a free-standing anode has always been a research hotspot [109,144], and will provide a simpler industrial process and greater commercial potential.

In conclusion, HC materials can be optimized by pre-treatment and self-supporting structure design, which can greatly improve electrochemical performance, reduce industrial cost, and simplify the process of making the full-cell. In addition to material optimization, electrolyte design and selection are of great significance. A large number of comparative experiments are designed to select the appropriate electrolyte system to improve battery performance. Furthermore, theoretical studies have confirmed the influence of electrolyte on electrode material properties [145]. For the ether-based electrolyte mentioned above, the fast sodium storage kinetics of HC in ether-based electrolyte can be attributed to three aspects: (i) The migration rate of Sodium-ions in ether-based electrolyte is higher than that of carbonates; (ii) the SEI film formed by the decomposition of ether-based electrolyte has more inorganic components, is thinner, and exhibits smaller interfacial impedance; and (iii) solvated Na^+^ in ether-based electrolyte not only has a higher ionic diffusion coefficient, but also causes more pseudo-capacitance behaviors. In a word, for the design of different HC materials, attention should also be paid to the matching of an electrolyte. Optimization of such battery conditions plays an important role in improving the performance of the full battery. Low-cost, safe, and stable electrolyte systems, additives, and non-binder anode materials are also strategic conditions for the industrialization and commercialization of SIBs.

## 5. Summary and Outlook

SIBs are regarded as safe, stable, low-cost and hold great feasibility for mass-produced energy storage systems. They are promising alternatives to LIBs. HCs are popular anode materials for industrialization with many advantages. Although the research surrounding an SIB and its anode carbon-based materials has lasted for decades, its commercialization has taken a step forward in recent years [146]. Considering the needs of industry, researchers need to find the right balance between cost and performance in order to be on a par with the LIBs that have been used for many years. To better find the optimal selection of an SIB carbon-based anode material, it is necessary to start with the perspective of its working mechanism, understand its sodium storage behavior from both microstructure and macroscopic properties, and then explore optimization and modification strategies on this premise. Herein, the development of HC materials in recent years, especially the sodium storage mechanism and the modification and optimization of an HC anode, are critically reviewed. On the one hand, research on the mechanism of sodium storage in HC materials is constantly improving and developing along with the controversy. As electrochemical analysis methods become more advanced, more and clearer methods are available to observe and clarify sodium storage behavior. Ex situ XRD, Raman, TEM, SAXS, XPS, and EPR are utilized to reveal the microscopic changes in the sodium storage process, and an efficient galvanostatic-potentiostatic mode was used to explore the kinetics of sodium storage in response to large currents [147]. With the improvement of research methods, revealing the mechanism of HC sodium storage is of great significance to produce SIB anode materials with high capacitive properties. On the other hand, HC can be modified by means of heteroatom-doping, structural regulation and surface modification, and conditions can be optimized from the perspective of an electrolyte, additive and binder to enhance the electrochemical performance of SIBs using a HC anode.

HC material is the most potential SIB anode material for commercialization. Although many contributions have been made to improve their performance, an HC-based full battery still has a bottleneck that needs to be broken. For example, compared with LIBs, there is still a significant difference in volume energy density, which limits the application of SIBs in many occasions with strict energy requirements, such as in the new energy vehicles (NEVs) field. It needs to explore more advantages of SIBs besides the cost advantages to optimize their market prospects [148]. From an optimistic perspective, a SIB, especially a full battery with a carbon base anode, has a good application trend in a large-scale power grid that would benefit from its low-cost, security and stability [149]. In the field of power grid energy storage, more than ten companies in the world have started to commercialize SIB technology. In 2019, a 30 kW/100 kWh SIB energy storage system was successfully installed in China [150]. In summary, the application prospect of HC materials in SIBs are promising, given the many optimistic and exciting research results that have been reported in recent years. “Beyond the LIB” is the original intention of the research and development of SIBs and is in a continuing process of development. To cater for the various energy demands, from small electronic products to large-scale power grid storage, an HC anode plays an integral role, which could push battery technology into a low-cost, high-performance, safer, and more stable direction. It will continue to receive the attention of enterprises and researchers.

## Figures and Tables

**Table 1:** Different kinds of anode materials in SIBs.

	Anode Material	Current Rate	Electrochemical Performance * (Reversible Capacity, Cycles, Capacity Retention)	Ref.
Titanium-based	NaTiOPO_4_	0.1 C	180 mAh g^−1^, /, /	[15]
	Ti_2_(SO_4_)_3_	0.1 C	120 mAh g^−1^, 15, 77.5%	[16]
Metal oxides	Na_2_Ti_3_O_7_	0.04 C	~200 mAh g^−1^, /, /	[17]
	α-MoO_3_	0.1 C	100 mAh g^−1^, 500, 55%(0.2 C)	[18]
Metallic composite	a-TiO_2-x_/Sb	100 mA g^−1^	591.9 mAh g^−1^, 200, 96.4%(1 A g^−1^)	[19]
	SiC–Sb–C	100 mA g^−1^	595 mAh g^−1^, 100, 80.7%	[20]
	Ti_3_C_2_T_x_/ SnP	0.2 A g^−1^	587 mAh g^−1^, 1000, 91.2%	[21]
Organic	Na_2_C_8_H_4_O_4_	0.1 C	258 mAh g^−1^, 50, 74.4%	[22]
	Organic sodium carboxylate salts	40 mA g^−1^	>200 mAh g^−1^(full-cell), 50, /	[23]
Carbon−based	Carbon black	C/75	121 mAh g^−1^, /, /	[24]
	HC	0.1 C	300.6 mAh g^−1^, 100, 98.1%	[26]
	Reduced graphene oxide	0.2 C	174.3 mAh g^−1^, 1000, 80.9%	[27]
	Soft carbon	20 mA g^−1^	232 mAh g^−1^, 40, 98.1%	[28]

* Half-cell electrochemical measurements vs. Na/Na^+^.
